# Cross-Jurisdictional Data Exchange Impact on the Estimation of the HIV Population Living in the District of Columbia: Evaluation Study

**DOI:** 10.2196/publichealth.9800

**Published:** 2018-08-13

**Authors:** Auntre D Hamp, Rupali K Doshi, Garret R Lum, Adam Allston

**Affiliations:** ^1^ HIV/AIDS, Hepatitis, STD, Tuberculosis Administration District of Columbia Department of Health Washington, DC United States; ^2^ Department of Epidemiology and Biostatistics The George Washington University Washington, DC United States

**Keywords:** HIV, surveillance, data sharing, public health, cross-jurisdictional

## Abstract

**Background:**

Accurate HIV surveillance data are essential to monitor trends to help end the HIV epidemic. Owing to strict policies around data security and confidentiality, HIV surveillance data have not been routinely shared across jurisdictions except a biannual case-by-case review process to identify and remove duplicate cases (Routine Interstate Duplicate Review, RIDR). HIV surveillance estimates for the District of Columbia (DC) are complicated by migration and care seeking throughout the metropolitan area, which includes Maryland and Virginia. To address gaps in HIV surveillance data, health departments of DC, Maryland, and Virginia have established HIV surveillance data sharing agreements. Although the Black Box (a privacy data integration tool external to the health departments) facilitates the secure exchange of data between DC, Maryland, and Virginia, its previous iterations were limited by the frequency and scope of information exchanged. The health departments of DC, Maryland, and Virginia engaged in data sharing to further improve HIV surveillance estimates.

**Objective:**

This study assessed the impact of cross-jurisdictional data sharing on the estimation of people living with HIV in DC and reduction of cases in the RIDR process.

**Methods:**

Data sharing agreements established in 2014 allowed for the exchange of HIV case information (eg, current residential address) and laboratory information (eg, test types, result dates, and results) from the enhanced HIV/AIDS Reporting System (eHARS). Regular data exchanges began in 2017. The participating jurisdictions transferred data (via secure file transfer protocol) for individuals having a residential address in a partnering jurisdiction at the time of HIV diagnosis or evidence of receiving HIV-related services at a facility located in a partnering jurisdiction. The DC Department of Health compared the data received to DC eHARS and imported updated data that matched existing cases. Evaluation of changes in current residential address and HIV prevalence was conducted by comparing data before and after HIV surveillance data exchanges.

**Results:**

After the HIV surveillance data exchange, an average of 396 fewer cases were estimated to be living in DC each year from 2012 to 2016. Among cases with a residential status change, 66.4% (1316/1982) had relocated to Maryland and 19.8% (392/1982) to Virginia; majority of these had relocated to counties bordering DC. Relocation in and out of DC differed by mode of transmission, race and ethnicity, age group, and gender. After data exchange, the volume of HIV cases needing RIDR decreased by 74% for DC-Maryland and 81% for DC-Virginia.

**Conclusions:**

HIV surveillance data exchange between the public health departments of DC, Maryland, and Virginia reduced the number of cases misclassified as DC residents and reduced the number of cases needing RIDR. Continued data exchanges will enhance the ability of DC Department of Health to monitor the local HIV epidemic.

## Introduction

Both the National HIV/AIDS Strategy released by the White House Office of National AIDS Policy in 2010 [1] and the 2016 District of Columbia 90/90/90/50 Plan to End the HIV Epidemic by 2020 [2] include key goals and outcome measures that depend on having an accurate population estimate of the number of individuals diagnosed and living with HIV. The four main aims of the District of Columbia (DC) Plan included the following: 90% knowing their HIV status, 90% engagement in HIV care, 90% viral suppression among those who enter care, and 50% reduction in new HIV diagnoses by 2020. Because the National HIV/AIDS Surveillance System (NHSS) aims to document all people diagnosed with HIV in the United States, the system is uniquely poised to provide a foundational denominator for these outcomes. Participants in NHSS consist of state and local health departments with public health authority to collect data on people living with HIV (PLWH). Thus, it is incumbent upon the participants of NHSS to provide the most up-to-date HIV prevalence data possible. In addition, having up-to-date HIV surveillance data would make data-to-care strategies, which use surveillance data to identify PLWH who are not achieving optimal health outcomes, more efficient [3].

NHSS supports the systematic collection of HIV and AIDS cases in the United States by 59 jurisdictions (states and territories), including the DC [4]. The data collected in NHSS are utilized to monitor the HIV epidemic, inform care, treatment, and prevention efforts and enable local health departments to report to the United States Centers for Disease Control and Prevention (CDC). Data are collected and maintained on local instances of the NHSS’s data collection system, the enhanced HIV/AIDS Reporting System (eHARS), which is a browser-based application. In addition to HIV-related diagnostic and clinical laboratory data, demographic data, risk information, treatment facility, and residential address are collected from health care providers and stored in eHARS. Each NHSS participant shares deidentified data with CDC monthly [5].

The Routine Interstate Duplicate Review (RIDR) process facilitates the identification and exchange of information across jurisdictions concerning individuals diagnosed with HIV who are documented in the eHARS databases of different jurisdictions. Although the main purpose of this process is deduplication, resident addresses may be exchanged, allowing jurisdictions to further refine their local estimates of PLWH. Certain authorized personnel at the state, county, and local health departments are permitted to discuss cases if there is an indication that the individual may have been in another state’s surveillance system. The Council of State and Territorial Epidemiologists provides a platform for jurisdictions to maintain an up-to-date list of the personnel identified to conduct RIDR.

Migration and population growth have challenged the understanding of who is living with HIV in DC. The US Census Bureau reported that between 2010 and 2016, the population of DC increased by an estimated 79,447 (13.2%) persons, and the Washington-Arlington-Alexandria Metropolitan Statistical Area population increased by an estimated 525,745 (8.8%) persons [6]. In addition to overall growth, according to the American Community Survey, between 2011 and 2015, approximately 24,530 persons moved out of DC to the surrounding counties of Prince George’s and Montgomery County, Maryland, Arlington and Fairfax County, Virginia, and Alexandria [7], and the racial majority of those who moved out of DC to those jurisdictions were black at 44.3% (10,868/24,530). The vast majority of black persons and Hispanic and Latino persons who left DC moved to Prince George’s County, Maryland, whereas the majority of the white persons who left DC moved to Montgomery County, Maryland. The overall population shifts make understanding the migration patterns of PLWH in the DC metropolitan area more challenging.

Residential address information is collected by NHSS, but it may not be updated beyond the initial case report collected at the time of HIV diagnosis. A lack of current residential addresses can stymie data-to-care efforts, which utilize residential address to re-engage people out of care; surveillance epidemiologists have found that the bulk of the effort is spent on updating addresses in eHARS, increasing the time to re-engagement [8]. Based on data in DC eHARS, PLWH may appear to be out of care but could have moved to a nearby county outside of DC and switched their care to a non-DC health care provider.

In 2013, the health departments of DC, Maryland, and Virginia met with Georgetown University to discuss the concept of sharing data across jurisdictions to expand the scope and timeliness of HIV surveillance data. By 2014, the three jurisdictions had agreed to share HIV surveillance data with each other and executed data sharing agreements (DSAs). DSAs included elements such as the frequency of sharing data, what variables would be shared, data security measures, and the format in which data would be transmitted. In 2014, National Institutes of Health funded Georgetown University to conduct a pilot study on a privacy sharing device for disease surveillance data known as the Black Box, in which the three jurisdictions participated. The Black Box pilot-tested a proof of concept that an encrypted, intermediary technology could receive surveillance data from the three health departments and securely report the probability of matches back to each jurisdiction. The pilot was successful in identifying multiple matches across the jurisdictions [8]. After seeing the success of the Black Box pilot and building upon the trust that was built during the setup of the Black Box pilot, the health departments of DC, Maryland, and Virginia recognized the need for more variables and routine exchanges of data to occur separately from relying upon the Black Box technology.

Starting in 2016, the health departments of DC, Maryland, and Virginia began to hold monthly conference calls that focused on the implementation of a routine exchange of HIV surveillance data (independent of the Black Box) between the three jurisdictions. Goals of the data exchange included the following: increasing information utilized to assess the HIV care continuum through the exchange of laboratory data; increase the ability to deduplicate cases through the exchange of personally identifiable surveillance data (ie, first name, last name, and date of birth); and increase the accuracy of the estimation of PLWH in DC by utilizing current residential information received through the data exchange. The objectives of this evaluation were to assess the impact of cross-jurisdictional data sharing on the estimation of PLWH in DC and reduction of cases needing review in the RIDR process.

## Methods

### Cross-Jurisdictional Operations Coordination and Governance Structure

Discussions about the concept of cross-jurisdictional exchanges of HIV data between DC Department of Health, Maryland Department of Health, and Virginia Department of Health began in January 2013. At the outset, all three jurisdictions needed substantial organizational and leadership buy-in and support from the general counsels to execute DSAs. In addition, following the execution of DSAs, key stakeholders in the surveillance divisions provided more nuanced input to plan for implementation. Beginning in 2016, the three jurisdictions established the DC, Maryland, and Virginia Regional (DMV) HIV Surveillance group, which comprised the leadership of the three jurisdictions’ HIV surveillance units, epidemiologists, eHARS data managers, and case surveillance coordinators. The group scheduled monthly calls to plan and review progress. In between the monthly calls, a subcommittee of epidemiologists from each health department developed the specific procedures of the data exchange, including the data elements to be shared, the frequency of exchanges, and validation of results. Variables chosen to be part of the exchange included case information, HIV diagnostic testing, viral load, CD4 results, and genotype sequence data (Multimedia Appendix 1).

### Data Extraction and Exchange Procedures

Each jurisdiction used the same SAS v9.4 (SAS Institute, Inc, Cary, North Carolina, USA) code to extract data from their respective instances of eHARS. The data files were encrypted and uploaded to a secure file transfer protocol site hosted by Maryland Department of Health. The epidemiologists who conducted the data extraction notified the respective jurisdictions of the uploaded data and provided encryption passwords to designated personnel. Upon receipt of the shared files, each jurisdiction assessed data quality and communicated about data gaps and inconsistencies.

The initial data exchange included data entered into eHARS from January 1, 2015, to March 31, 2017. The data sent by jurisdictions included cases for which the state listed for residence at HIV diagnosis, residence at AIDS diagnosis, HIV diagnosing facility, AIDS diagnosing facility or laboratory facility state matched the receiving jurisdiction. During this initial exchange, DC Department of Health received 56,451 laboratory results from Maryland Department of Health and 15,090 from Virginia Department of Health. DC Department of Health provided Maryland Department of Health with 82,683 laboratory results and provided Virginia Department of Health with 97,467 (Table 1).

**Table 1:** Laboratory results exchanged by the jurisdictions.

Results received by and sent to jurisdictions sent by DC^a^	Jurisdiction (n)	
	Virginia	Maryland
Laboratory results received	15,090	56,451
Laboratory results sent	97,467	82,683

^a^DC: District of Columbia.

**Table 2:** Data matching criteria.

Match Level	Matching criteria
Match 1	If First Name, Last Name, Date of Birth
Match 2	Else if, First Name (First 6 Letters), Last Name, Date of Birth
Match 3	Else if, Last Name (First Letter), Last Name (Letters 3 through 8), First Name (Letters 2 through 8), Date of Birth
Match 4	Else if, Last Name (First Letter), Last Name (Letters 3 through 8), First Name (Letters 2 through 8), Birth Month, Birth Year
Match 5	Else if, Last Name (First Letter), Last Name (Letters 3 through 8), First Name (Letters 2 through 8), Birth Day, Birth Year
Match 6	Else, if Last Name, First Name (Letters 1 through 2), Date of Birth
Match 7	Else, if Last Name (Letters 1 through 3), First Name (Letters 1 through 3), Date of Birth
Match 8	Else if, Last Name (Letters 1 through 4), First Name (Letters 1 through 4), Birth Year
Match 9	Else if, First Name (Letters 1 through 3), Last Name (Letters 1 through 3), Birth Month, Birth Year
Match 10	Else if, First Name (Letters 1 through 3), Last Name (Letters 1 through 3), Birth Day, Birth Year
Match 11	First Name (Letters 1 through 3), Last Name (Letters 1 through 3), Birth Month, Birth Year

### Data Matching Procedures

Each jurisdiction used their own matching procedures and algorithms to assess whether the person-level data received during the exchange matched persons currently in their eHARS system. DC Department of Health used an 11-key algorithm in SAS (Table 2) to match incoming data from exchange with existing persons in DC eHARS. The first match key assessed exact matches, which consisted of first name, last name, and date of birth, whereas the other match key criteria allowed for slight variation in how the surveillance information may have been recorded.

### Estimating People Living With HIV in the District of Columbia

Calculations of the number of PLWH vary by jurisdiction. For the purpose of this study, DC estimated the number of PLWH based on evidence of a DC residential address within the previous 5 years and having associated laboratory data present within the same time period; for example, when estimating PLWH for 2016, persons with a DC address within the past 5 years who also had laboratory records between 2012 and 2016 would be included in the estimate. This is consistent with how the DC prevalence estimate was presented at the Annual Epidemiology and Surveillance Report from DC [9]. DC recognizes that this may differ from HIV prevalence estimates published by CDC; however, owing to the high amount of population movement in and out of DC, it is believed this would produce a more accurate estimate.

### Routine Interstate Duplicate Review

RIDR is a process coordinated by CDC, in which a Soundex match is conducted on national data to identify potential duplicates within the system. Soundex is a coded index associated with how a name sounds versus how a name is spelled. Jurisdictions receive lists semiannually and typically correspond with one another by telephone to ascertain whether or not the persons identified are same or different. Staff from each jurisdiction record a duplicate review status in eHARS and exchange new current residential addresses and recent laboratory information [5]. Updating these data enable jurisdictions to identify persons who have moved between jurisdictions. Information received from the data exchange was utilized to update RIDR information on matched persons without the need to conduct manual RIDR processes.

### Data Analysis

The current residential address is calculated and updated in eHARS from incoming case reports and laboratory data obtained from health care providers, laboratories, or other health departments.

Analytic datasets were derived before and after uploading exchanged data from Maryland and Virginia into DC eHARS; these became the pre-exchange and postexchange datasets. The main outcomes of this analysis were the change in the estimate of PLWH in DC and the reduction in the number of cases needing RIDR between DC and Maryland and between DC and Virginia after the data exchange.

## Results

### Changes in Residential Jurisdiction

**Figure 1:**
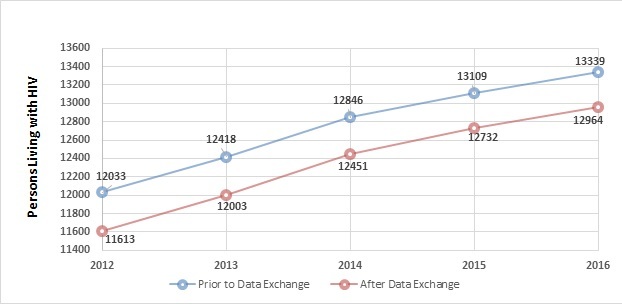
People living with HIV in Washington, District of Columbia (DC), 2012-2016, before and after HIV surveillance data exchange between DC, Maryland, and Virginia.

After the HIV surveillance data exchange between DC and Maryland and Virginia, there were 396 fewer persons estimated to be living with HIV in DC each year between 2012 and 2016, as seen in Figure 1. There was an average −3.1% difference (pre-exchange versus postexchange) over this time period.

**Figure 2:**
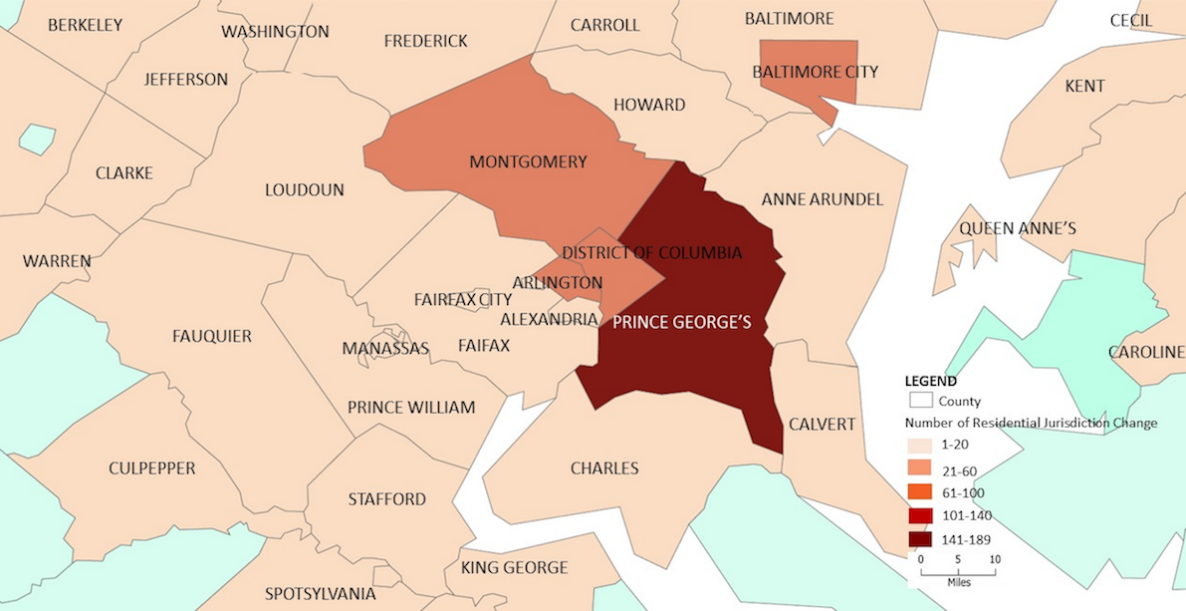
Distribution of persons with a change in residential jurisdiction, 2016.

**Table 3:** Updated current state of residence after HIV surveillance data exchange between the District of Columbia, Maryland, and Virginia, by Jurisdiction, 2016.

Residential state	People living with HIV with a change in residential jurisdiction (N=426), n (%)
California	1 (0.2)
District of Columbia	43 (10.1)
Delaware	1 (0.2)
Maryland	284 (66.7)
Mississippi	1 (0.2)
North Carolina	1 (0.2)
New Jersey	1 (0.2)
New York	7 (1.6)
Ohio	1 (0.2)
Oklahoma	1 (0.2)
Pennsylvania	1 (0.2)
Texas	1 (0.2)
Virginia	83 (19.5)

Of the 426 persons who were found to have a non-DC residence in 2016, the majority had an address in Maryland (284/426, 66.7%) or Virginia (83/426, 19.5%). Most of the individuals who appear to have moved out of DC were in one of the adjacent counties: Prince George’s County, Maryland (n=138), Montgomery County, Maryland (n=34), and Arlington County, Virginia (n=23). Figure 2 geospatially depicts the persons whose current residence changed owing to information received in the data exchange with most people shown to be living closely along the border of DC. It was also found that 43 people changed their residence from either Maryland or Virginia to DC (Table 3).

Most people with a change in residential jurisdiction were male. Males represented 74.7% (212/284) of those with a new residential address in Maryland and 79.5% (66/83) of persons with a new address in Virginia. Among those whose residential jurisdiction changed to DC, 83.7% (36/43) were male. Just under 50% of migrants to Maryland (137/284, 48.2%) had a mode of transmission of men who have sex with men (MSM) or MSM and injection drug use (MSM/IDU). Similarly, when assessing migrants by mode of transmission, MSM and MSM/IDU represented the majority of persons who migrated to Virginia (47/83, 56.6%). When assessing those with an evidence of a change in residency to either of the three jurisdictions, among those with a mode of transmission of IDU, there were relatively similar distributions by jurisdiction at 9.3% (4/43) to DC, 8.5% (24/284) to Maryland, and 8.4% (7/83) to Virginia. Similar results were found when assessing heterosexual contact, wherein 29.9% (85/284) of persons with a new Maryland residence, 26.5% (22/83) of persons with a new Virginia address, and 23.3% (10/43) of persons with a new DC address had heterosexual contact as a mode of transmission. Changes of address among racial or ethnic categories showed significant differences with black persons or African Americans making up a larger percentage of those who migrated to DC (31/43, 72.1%) and Maryland (234/284, 82.4%) compared with Virginia (48/83, 57.8%). Additionally, Hispanic and Latino persons represented a higher proportion of persons moving to Virginia (12/83, 14.5%) than those who moved to DC (4/43, 9.3%) or Maryland (19/284, 6.7%). White persons represented a smaller proportion of those who moved to Maryland (21/284, 7.4%) when compared with DC (8/43, 18.6%) and Virginia (20/83, 24.1%). When looking at age groups, persons with a residential change into DC were more likely to be over 40 years old, whereas persons aged between 25 and 39 years represented the majority of persons with a residential change to Maryland (148/283, 52.2%) and Virginia (48/83, 57.8%) (Table 4).

**Table 4:** Demographic characteristics of people living with HIV with a change in residential jurisdiction, by state, 2016.

Characteristics		People living with HIV with a change in residential jurisdiction			People living with HIV in DC^a^ (N=12,964), n (%)
		DC (N=43), n (%)	Maryland (N=284), n (%)	Virginia (N=83), n (%)	
**Gender**					
	Female	7 (16.3)	70 (24.6)	15 (18.1)	3395 (26.2)
	Female to male	0 (0.0)	2 (0.7)	0 (0.0)	72 (0.6)
	Male	36 (83.7)	212 (74.6)	66 (79.5)	9352 (72.1)
	Male to female	0 (0.0)	0 (0.0)	2 (2.4)	145 (1.1)
**Mode of transmission**					
	MSM^b^	24 (55.8)	127 (44.7)	42 (50.6)	5650 (43.6)
	IDU^c^	4 (9.3)	24 (8.5)	7 (8.4)	1372 (10.6)
	MSM/IDU	0 (0.0)	10 (3.5)	5 (6.0)	404 (3.1)
	Heterosexual contact	10 (23.3)	85 (29.9)	22 (26.5)	3689 (28.5)
	Risk not identified	4 (9.3)	31 (10.9)	7 (8.4)	1703 (13.1)
	Other^d^	1 (2.3)	7 (2.5)	0 (0.0)	146 (1.1)
**Race or ethnicity**					
	White	8 (18.6)	21 (7.4)	20 (24.1)	2076 (16.0)
	Black	31 (72.1)	234 (82.4)	48 (57.8)	9670 (74.6)
	Hispanic	4 (9.3)	19 (6.7)	12 (14.5)	884 (6.8)
	Other^e^	0 (0.0)	10 (3.5)	3 (3.6)	334 (2.6)
**Age group**					
	0-12	1 (2.3)	5 (1.8)	0 (0.0)	22 (0.2)
	13-19	2 (4.7)	17 (6.0)	4 (4.8)	60 (0.5)
	20-24	7 (16.3)	48 (16.9)	11 (13.3)	331 (2.6)
	25-29	7 (16.3)	47 (16.5)	21 (25.3)	908 (7.0)
	30-39	9 (20.9)	101 (35.6)	27 (32.5)	2452 (18.9)
	40-49	12 (27.9)	49 (17.3)	16 (19.3)	2963 (22.9)
	50-59	5 (11.6)	15 (5.3)	4 (4.8)	3957 (30.5)
	>=60	0 (0.0)	2 (0.7)	0 (0.0)	2268 (17.5)
	Missing	0 (0.0)	0 (0.0)	0 (0.0)	3 (0.0)

^a^DC: Dictrict of Columbia.

^b^MSM: men who have sex with men.

^c^IDU: injection drug use.

^d^Other mode of transmission includes perinatal transmission, hemophilia, blood transfusion, and occupational exposure (health care workers).

^e^Other race includes mixed-race individuals, Asians, American Indians, Native Hawaiians, Pacific Islanders, and unknown races.

**Table 5:** January 2017 Routine Interstate Duplicate Review (RIDR) cases resolved by the District of Columbia, Maryland and Virginia region HIV surveillance data exchange.

Total HIV cases identified by Centers for Disease Control and Prevention	Maryland (N=171), n (%)	Virginia (N=82), n (%)
RIDR cases resolved by data exchange	127 (74.3)	67 (81.7)
Remaining HIV cases needing RIDR	44 (25.7)	15 (18.3)

### Changes in Routine Interstate Duplicate Review

RIDR activities are typically conducted through the exchange of case information via the telephone. Telephonic RIDR resolution activities between DC and Maryland and DC and Virginia were not conducted prior to the data exchange. The HIV surveillance data exchange among DC, Maryland, and Virginia allowed for RIDR information to be exchanged electronically and decreased the number of cases identified by RIDR needing manual resolution by 74.3% (127/171) between DC and Maryland and by 81.7% (67/82) between DC and Virginia (Table 5). This has had a significant impact in reducing the workload of health department staff in all three jurisdictions. Additionally, the data sharing process has contributed to an overall reduction in the number of persons needing resolution between the three jurisdictions because duplicates were identified earlier than with the biannual RIDR process. For the July 2017 RIDR list produced by CDC, DC Department of Health saw a reduction in resolution case volume of 61.4% between DC and Maryland and 43.9% between DC and Virginia compared with the January 2017 RIDR list.

## Discussion

Although the overall population estimates between 2012 (635,630) and 2016 (684,336) in DC increased by 7.1% [6], based on our analysis, between 375 and 420 PLWH migrated out of DC each year over the past five years. This represents a −3.1% change in PLWH in DC over this time period. Although this percent decrease is relatively small, the absolute number of persons deemed to be living in a different jurisdiction represents a significant amount of surveillance personnel effort that would have been exerted in re-engagement in care efforts. There are many factors that may contribute to migration in and out of DC, but they are beyond the scope of this paper. However, it is interesting to note that the majority of individuals diagnosed with HIV who moved out of DC stayed within the surrounding counties (Prince George’s County and Montgomery County in Maryland, and Fairfax County, Arlington County, and Alexandria City in Virginia), which are part of the DC Ryan White Part A Eligible Metropolitan Area. Individuals who are Ryan White-eligible would still be able to access services offered in the Part A geographic area. However, other services they may need, such as Medicaid or the AIDS Drug Assistance Program, would need to be accessed from their new residential jurisdiction because they are distributed by states only.

We demonstrated a significant reduction in cases needing to be resolved via the labor-intensive RIDR process after the implementation of the data exchange. Cross-jurisdictional HIV surveillance data exchange is feasible and could be of great benefit to other areas of the United States where there are substantial movement across states or jurisdictions. The protocol used in the DMV HIV surveillance data exchange has made DC Department of Health HIV surveillance operations more efficient.

Testing and treatment methods with new advanced biomedical interventions have become the cornerstone of strategies to reduce new HIV infections. The 90/90/90/50 Plan to End the HIV Epidemic by 2020 in the District of Columbia set goals to ensure that 90% of persons with HIV know their status, 90% of persons diagnosed with HIV are retained in HIV care and treatment, and 90% of persons on treatment are virally suppressed, resulting in a 50% reduction in cases by 2020 over the baseline year of 2015 [10]. To meet these measures, a robust surveillance system is needed to identify new cases of HIV and monitor HIV care markers with an accurate denominator. The DMV HIV surveillance data exchange has enabled DC Department of Health to more accurately identify persons residing within the jurisdiction to better track and assess health outcome measures.

Data-to-care efforts in DC have focused on locating PLWH who appear to be out of care based on clinical and surveillance data [11]. Prior to the DMV HIV surveillance data exchange, individuals who moved out of DC may have appeared to be out of care, but they relocated their residence and health care. Data exchange resulted in updated residential addresses and reduced the number of people potentially needing re-engagement in care. The updated address data will significantly assist the data-to-care efforts in DC with more accurate location information of people who may be in need of outreach, re-engagement, treatment adherence, and other enabling or support services. The data exchange also updated laboratory data, which is critical to understanding who may need more intensive public health interventions, such as individuals with low CD4 cell counts or high viral load levels. The use of updated residential address and laboratory data in this way affirms the utility of collecting this information from PLWH. Future analyses may include pre- and postexchange comparison of engagement in care and viral suppression among PLWH in DC.

Data exchange was limited to three states with moderate HIV prevalence. The DMV HIV surveillance data exchange may be enhanced by exchanging data with other nearby states, such as New York, New Jersey, Pennsylvania, and North Carolina, with high levels of RIDR overlap with cases in DC. This was explored in a separate project (Black Box RIDR Resolution project) funded by CDC, in which additional states participate to identify potential matched cases in a secure and confidential manner, and the recently funded CDC-RFA-PS18-1805-Secure Data Sharing Tool awarded to Georgetown University. Additional limitations include in the validity of the accuracy of the matching algorithm. The 11-key matching algorithm was validated to be extremely accurate at the higher levels (match levels 1-4), although there is potential for mismatching at the lower levels (match levels 6-11).

The DMV HIV surveillance data exchange has demonstrated that conducting standardized matches of data across jurisdictions is feasible and provides timely resolution of duplicate cases that might otherwise require time-intensive, one-to-one conversations between health department staff. Other states, particularly jurisdictions in which PLWH may seek care across jurisdictional boundaries, may benefit from pursuing DSAs to conduct HIV surveillance data exchanges. More accurate epidemiologic data may be used for improving funding decisions around care and prevention programs, particularly in areas with significant levels of population movement and migration.

## Appendix

Multimedia Appendix 1 Detailed variables extracted from eHARS.

## Abbreviations

CDC: Centers for Disease Control and Prevention
DC: District of Columbia
DMV: District of Columbia, Maryland and Virginia region
DSA: data sharing agreement
eHARS: enhanced HIV/AIDS Reporting System
IDU: injection drug use
MSM: men who have sex with men
NHSS: National HIV/AIDS Surveillance System
PLWH: people living with HIV
RIDR: Routine Interstate Duplicate Review
