# Supplementary material for: Cross-Jurisdictional Data Exchange Impact on the Estimation of the HIV Population Living in the District of Columbia: Evaluation Study
Source: JMIR Public Health Surveill. 2018 Aug 13;4(3):e62. doi: 10.2196/publichealth.9800 (PMC6111149; doi:10.2196/publichealth.9800)
Supplement: Multimedia Appendix 1 [file publichealth_v4i3e62_app1.pdf]

I. Detailed variables extracted from eHARS

**eHARS Variable Name**	**Variable Description**
aids_rep_dt	AIDS Report Date
birth_sex	Birth Sex
cur_state_cd	Current State Code
Dob	Date of Birth
dod	Date of Death
death_state_cd	Death State
dx_status	Diagnosis Status
ehars_uid	eHARS Unique Identifier
ethnicity1	Ethnicity (Hispanic/Latino; Not Hispanic/Latino)
expo_categ	Exposure Category
hiv_aids_dx_dt	HIV/AIDS Diagnosis Date
hiv_aids_rep_dt	HIV/AIDS Report Date
hiv_dx_dt	HIV Diagnosis Date
aids_dx_dt	AIDS Diagnosis Date
hiv_rep_dt	HIV Report Date
race	Race
report_state_cd	Reporting Jurisdiction State
stateno	Stateno
trans_categ	Transmission Category
vital_status	Vital Status
cur_city_fips	Current City FIPS
cur_city_name	Current City Name
cur_country_cd	Current Country Code
cur_country_usd	Current Country

cur_county_fips	Current County FIPS
cur_county_name	Current County Name
cur_phone	Current Phone
cur_state_cd	Current State Code
cur_address_dt	Current Address Date
cur_street_address1	Current Street Address
cur_street_address2	Current Street Address (additional)
cur_zip_cd	Current Zip code
current_gender	Current Gender
first_name	First Name
last_name	Last Name
hf_city_fips	HIV Facility City FIPS
hf_city_name	HIV Facility City Name
hf_country_cd	HIV Facility Country Code
hf_country_usd	HIV Facility Country
hf_county_fips	HIV Facility County FIPS
hf_county_name	HIV Facility County Name
hf_facility_type_cd	HIV Facility Type Code
hf_facility_uid	HIV Facility Unique Identifier
hf_fax	HIV Facility Fax
hf_name1	HIV Facility Name
hf_name2	HIV Facility Name (additional)
hf_phone	HIV Facility Phone
hf_provider_first_name	HIV Facility Provider First Name
hf_provider_last_name	HIV Facility Provider Last Name
hf_provider_middle_name	HIV Facility Provider Middle Name
hf_provider_name_prefix	HIV Facility Provider Name Prefix

hf_provider_name_suffix	HIV Facility Provider Name Suffix
hf_provider_phone	HIV Facility Provider
hf_provider_uid	HIV Facility Provider
hf_setting_cd	HIV Facility Setting Code
hf_state_cd	HIV Facility Setting
hf_street_address1	HIV Facility Setting
hf_street_address2	HIV Facility Setting
hf_zip_cd	HIV Facility Setting
rsd_city_fips	Residence at Death City FIPS Code
rsd_city_name	Residence at Death City
rsd_country_cd	Residence at Death Country
rsd_country_usd	Residence at Death Country
rsd_county_fips	Residence at Death County FIPS
rsd_county_name	Residence at Death County
rsd_phone	Residence at Death Phone
rsd_state_cd	Residence at Death State
rsd_street_address1	Residence at Death Street Address
rsd_street_address2	Residence at Death Street Address (additional)
rsd_zip_cd	Residence at Death State
rsh_census_block_group	Residence at HIV Diagnosis State
rsh_census_congress_district	Residence at HIV Diagnosis State
rsh_census_group	Residence at HIV Diagnosis State
rsh_census_msa	Residence at HIV Diagnosis State
rsh_census_tract	Residence at HIV Diagnosis State
rsh_city_fips	Residence at HIV Diagnosis State
rsh_city_name	Residence at HIV Diagnosis State
rsh_country_cd	Residence at HIV Diagnosis State

rsh_country_usd	Residence at HIV Diagnosis State
rsh_county_fips	Residence at HIV Diagnosis State
rsh_county_name	Residence at HIV Diagnosis State
rsh_phone	Residence at HIV Diagnosis State
rsh_state_cd	Residence at HIV Diagnosis State
rsh_street_address1	Residence at HIV Diagnosis State
rsh_street_address2	Residence at HIV Diagnosis State
rsh_zip_cd	Residence at AIDS Diagnosis Zip Code
rsa_state_cd	Residence at AIDS Diagnosis State
af_state_cd	AIDS Facility State Code
af_name1	AIDS Facility Name
af_name2	AIDS Facility Additional Name
af_phone	AIDS Facility Phone
af_city_name	AIDS Facility City
af_provider_first_name	AIDS Facility Provider First Name
af_provider_last_name	AIDS Facility Provider Last Name
af_provider_middle_name	AIDS Facility Middle Name
af_provider_phone	AIDS Facility Phone
af_state_cd	AIDS Facility State Code
af_street_address1	AIDS Facility Street Address
af_street_address2	AIDS Facility Street Address (additional)
af_zip_cd	AIDS Facility Zip Code
test_recent_dt	Most Recent Test Date
rsa_census_msa	Residence at AID Diagnosis Census MSA
rsa_census_tract	Residence at AID Diagnosis Census Tract
rsa_city_fips	Residence at AID Diagnosis City FIPS
rsa_city_name	Residence at AID Diagnosis City Name

rsa_country_cd	Residence at AID Diagnosis Country Code
rsa_country_usd	Residence at AID Diagnosis Country
rsa_county_name	Residence at AID Diagnosis County Name
rsa_county_fips	Residence at AID Diagnosis County FIPS
rsa_phone	Residence at AID Diagnosis Phone
rsa_state_cd	Residence at AID Diagnosis State Code
rsa_street_address1	Residence at AID Diagnosis Street Address
rsa_street_address2	Residence at AID Diagnosis Street Address (additional)
rsa_zip_cd;	Residence at AID Diagnosis
document_uid	Document Unique Identifier
sample_id	Sample Indentifier
provider_uid	Provider Unique Identifier
specimen	Specimen Type
sample_dt	Sample Date
receive_dt	Lab Receive Date
manufacturer	Lab Manufacturer
clia_uid	CLIA Unique Identifier
lab_test_cd	Laboratory Test Code
result_interpretation	Laboratory Test Result Interpretation
result	Laboratory Test Result
result_units	Laboratory Test Laboratory Test
result_rpt_dt	Laboratory Test Result Report Date
Genotype_squence	Laboratory Test Genotype Sequence
comments	Laboratory Test Comments
starhs_sample_id	Laboratory Test STAHS
accession_number	Laboratory Test Accession Number
sreason	Laboratory Test

facility_uid	Laboratory Test Facility Unique Identifier
result_range_lower	Laboratory Test Result Lower Range
result_range_upper	Laboratory Test Result Upper Range
lab_seq	Laboratory Test Sequence
lab_facility_name1	Laboratory Facility Name
lab_facility_name2	Laboratory Facility Name (additional)
lab_facility_street_address1	Laboratory Facility Street Address
lab_facility_street_address2	Laboratory Facility Street Address (additional)
lab_facility_city_name	Laboratory Facility City Name
lab_facility_state_cd	Laboratory Facility State Code
lab_facility_zip_cd	Laboratory Facility Zip Code
lab_first_name	Laboratory Person First Name
lab_last_name	Laboratory Person Last Name
